# Real-Time Subjective and Physiological Responses During Dental Treatment: Temporal Characteristics of Skin Temperature and Electrodermal Activity

**DOI:** 10.3390/s26216695

**Published:** 2026-10-23

**Authors:** Jumpei Murakami, Tomomi Yamada, Takashi Sakamoto, Hiroshi Hanamoto, Kazunori Nozaki, Mikako Hayashi, Takashi Yamashiro

**Affiliations:** 1 Department of Special Needs Dentistry, Graduate School of Dentistry, The University of Osaka, 1-8 Yamadaoka, Suita City 565-0871, Osaka, Japan; sakamoto.takashi.dent@osaka-u.ac.jp; 2 Department of Restorative Dentistry and Endodontology, Graduate School of Dentistry, The University of Osaka, 1-8 Yamadaoka, Suita City 565-0871, Osaka, Japan; hayashi.mikako.hq@osaka-u.ac.jp; 3 Department of Dental Anesthesiology, Graduate School of Biomedical and Health Sciences, Hiroshima University, 1-2-3 Kasumi, Minami-ku, Hiroshima 734-8553, Japan; h-hanamoto@hiroshima-u.ac.jp; 4 Division for Oral Dental Informatics, The University of Osaka Dental Hospital, 1-8 Yamadaoka, Suita City 565-0871, Osaka, Japan; nozaki.kazunori.dent@osaka-u.ac.jp; 5 Department of Orthodontics, Graduate School of Dentistry, The University of Osaka, 1-8 Yamadaoka, Suita City 565-0871, Osaka, Japan; yamashiro.takashi.dent@osaka-u.ac.jp

**Keywords:** electrodermal activity, skin temperature, wearable sensors, dental treatment, autonomic responses, continuous monitoring, temporal dynamics

## Abstract

**Highlights:**

**What are the main findings?**

- Skin temperature and electrodermal activity showed changes that persisted into the post-treatment period, whereas subjective discomfort rapidly decreased after treatment completion.
- Temporal analyses provided additional information on response timing, persistence, and inter-individual variability beyond that obtained from phase-averaged analyses.

**What are the implications of the main findings?**

- Subjective and physiological responses showed different temporal characteristics, highlighting the importance of considering their temporal course when integrating these measures.
- Wearable sensor-based monitoring may help characterize the temporal course of physiological responses during dental treatment.

**Abstract:**

Dental treatment can induce stress and discomfort in patients; however, how physiological responses evolve over the course of treatment remains unclear. This study investigated physiological and real-time subjective responses during dental treatment, focusing on the temporal characteristics of fingertip skin temperature (TMP) and electrodermal activity (EDA) and their relationships with subjective responses. Fourteen participants were exposed to extraoral suction noise and subsequently underwent dental scaling. TMP and EDA were continuously recorded using wearable sensors, while real-time discomfort (RTD) was assessed using a continuous subjective rating device. Questionnaires were administered before and after treatment. TMP decreased during the Noise-only and Treatment phases; in most participants, the minimum temperature was observed after treatment completion. EDA increased during and after treatment, with inter-individual variability in peak timing and temporal course. Whereas RTD rapidly decreased after treatment completion, physiological responses persisted into the Post-treatment phase. The results suggest that TMP and EDA exhibit different temporal characteristics. Temporal analyses provided additional information on response timing, persistence, and inter-individual variability beyond that obtained from phase-averaged analyses. Although these findings are exploratory, they suggest the importance of examining whether quantitative measures derived from continuous physiological signals adequately represent the temporal course of the underlying physiological responses.

## 1. Introduction

Dental fear and anxiety are common, with an estimated global prevalence of approximately 15% among adults [1,2]. Psychological stress and discomfort during dental treatment may affect not only patient comfort but also safety, cooperation, and treatment outcomes. In particular, if stress is not appropriately recognized and managed during dental treatment, acute adverse events such as syncope, hypertension, and hyperventilation may occur [3,4,5].

In routine dental practice, communication between patients and dental professionals is often limited during intraoral procedures, making it difficult for patients to verbally express discomfort or distress [6,7]. Furthermore, individuals with intellectual disabilities, autism spectrum disorder, or communication disorders may be unable to adequately express discomfort or stress, requiring particularly careful management [8,9,10,11]. Therefore, real-time assessment of patients’ physiological and subjective responses during dental treatment is important for appropriate clinical decision-making and the provision of a safe treatment environment.

Dental treatment represents a distinct stress environment compared with general medical care. In addition to anxiety associated with the dental setting itself, patients are continuously exposed to multiple stressors, including dental treatment noise, invasive procedures, prolonged mouth opening, and various sensory stimuli throughout treatment [12,13,14,15]. As these stimuli change dynamically, physiological responses are also expected to vary over time. However, relatively few studies have continuously evaluated physiological responses associated with stress and discomfort during dental treatment and examined their temporal characteristics.

In recent years, physiological monitoring has attracted attention as a method for continuously and nonverbally evaluating emotional states without relying solely on subjective reports [16]. Various physiological parameters, including heart rate (HR) variability, electrodermal activity (EDA), skin temperature (TMP), respiratory rate, and blood pressure, have been investigated in relation to psychological stress using a range of physiological monitoring technologies [17,18,19,20,21,22]. In the dental field, studies have also examined EDA, TMP, HR, and blood pressure during dental procedures [15,23,24]. Infrared thermography has been used to measure changes in facial temperature in attempts to capture psychological stress and dental anxiety [23,25]. However, actual dental treatment involves multiple, dynamically changing stimuli, and it remains unclear how physiological responses evolve over the course of treatment in such clinical settings, including when they reach their peak, whether they persist after treatment, and how they recover. Furthermore, the temporal relationship between continuously changing subjective discomfort and physiological responses during dental treatment has not been fully investigated. Subjective discomfort is commonly assessed retrospectively or at discrete time points before and after treatment; however, such assessments may not reflect how discomfort changes moment by moment during treatment. Therefore, it remains unclear whether physiological responses show similar relationships with retrospectively assessed discomfort and with discomfort perceived in real time during treatment. Wearable sensors enable the acquisition of large amounts of continuous physiological data, from which numerous features can be derived for subsequent predictive modeling. However, predictive performance does not necessarily indicate what each input feature represents physiologically or which aspect of the temporal response it captures. For physiologically interpretable real-time monitoring, it is therefore important to examine continuous physiological signals in detail and determine what information is captured by measures of response magnitude, timing, persistence, and recovery during actual treatment.

Accordingly, this pilot study aimed to characterize the temporal changes in physiological responses during actual dental treatment and to examine their temporal relationships with real-time subjective discomfort, as well as their associations with subjective ratings obtained before and after treatment. This study was conducted as a preliminary step toward larger-scale clinical data collection and future predictive modeling, with the ultimate goal of developing physiologically interpretable real-time monitoring approaches for dental clinical settings.

## 2. Materials and Methods

### 2.1. Participants

Participants were recruited according to the following inclusion criteria: (1) presence of mild supragingival calculus deposition in the mandibular anterior region without signs of acute inflammation; (2) no history of cardiovascular disease; (3) no history of neurological disorders; and (4) no use of medications known to affect autonomic nervous system function. All participants received a detailed explanation of the study and provided written informed consent before participation. Participants were instructed to refrain from caffeine consumption for at least 8 h before the experiment; compliance was confirmed before data collection. A total of 15 adults participated in the study. One participant was excluded from the analysis because an irregular cardiac rhythm was detected during the experiment. Thus, 14 participants were included in the analyses. The 14 participants included in the analyses were aged 23–63 years (mean age, 30.8 years; 11 males and 3 females). Although eligibility criteria were applied to conditions considered likely to affect physiological measurements, age was not restricted to a narrow range because adults across a broad age range undergo dental treatment in clinical practice. For two of these participants, EDA and TMP data were unavailable because of inadequate sensor attachment; therefore, analyses involving EDA or TMP were performed using data from 12 participants, whereas analyses not involving these measures were performed using the available data from 14 participants.

This study was designed as an exploratory pilot study to examine the temporal characteristics of continuously recorded physiological responses during dental treatment and their temporal relationships with real-time subjective discomfort. Therefore, an a priori sample size calculation based on a specific primary outcome was not performed.

The study protocol was approved by the Ethics Committee of the Osaka University Graduate School of Dentistry (Approval No. R2-E22-2), and the study was conducted in accordance with the Declaration of Helsinki.

### 2.2. Experimental Design

All experiments were conducted in a dental treatment room under controlled lighting and background noise conditions. Air conditioning was not operated during the experimental protocol; this also helped reduce airflow and background noise. Room temperature was not recorded during the experiment. To minimize procedural variability, all dental procedures were performed by the same dentist using the same equipment. Supragingival scaling of the mandibular anterior teeth was performed using an ultrasonic scaler (Solfy, Morita Corp., Kyoto, Japan). The scaler output was set to level 2 on a 20-level scale, and scaling was performed under continuous water irrigation. Irrigation water was removed using the dental chair suction system.

In dental practice, an extraoral high-volume evacuator is widely used to reduce aerosol dispersion. To evaluate responses during exposure to the extraoral evacuator without intraoral dental treatment, the evacuator was positioned approximately 60 cm in front of the participant’s mouth and operated for 60 s before the dental procedure. The A-weighted equivalent continuous sound pressure level (*L*_Aeq_), measured near the participant’s ear using a precision sound level meter (LA-7000, Ono Sokki Co., Ltd., Yokohama, Japan) over the 60-s Noise-only exposure period, was approximately 80 dB.

**Figure 1:**
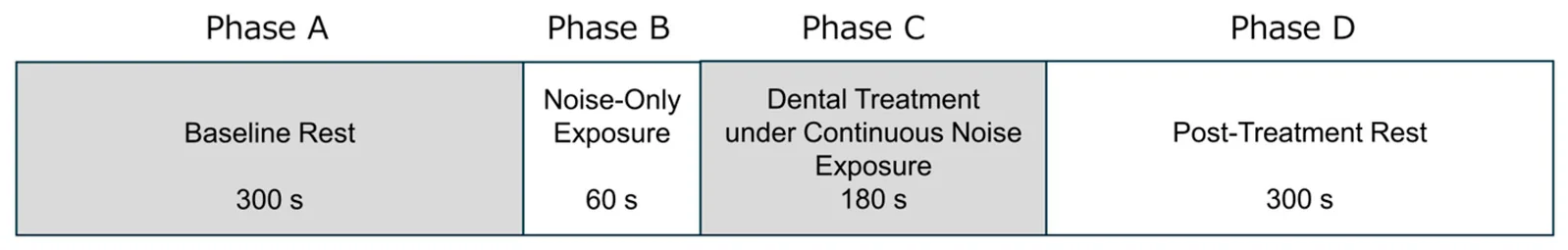
Experimental protocol comprising four sequential phases: Baseline Rest (Phase A), Noise-Only Exposure (Phase B), Dental Treatment under Continuous Noise Exposure (Phase C), and Post-Treatment Rest (Phase D).

The experimental protocol consisted of four phases: Baseline Rest (Phase A, 300 s), Noise-Only Exposure (Phase B, 60 s), Dental Treatment under Continuous Noise Exposure (Phase C, 180 s), and Post-Treatment Rest (Phase D, 300 s). For simplicity, these phases are hereafter referred to as Baseline, Noise-only, Treatment, and Post-treatment, respectively. The total experimental duration was approximately 14 min. During Phase B, only the extraoral evacuator was operated. During Phase C, ultrasonic scaling was performed while the evacuator remained in operation. During Phase D, participants remained in the supine position to allow recovery of physiological responses. The phases were conducted in a fixed sequence to reflect the temporal progression of a clinical dental procedure. The duration of scaling was fixed at 180 s for all participants, and any calculus remaining after this period was removed after completion of the experimental protocol. All physiological signals and real-time subjective discomfort ratings were continuously recorded throughout the experiment. An overview of the experimental protocol is shown in Figure 1.

### 2.3. Physiological Measurements

HR and systolic blood pressure (SBP) data were collected using a SOMNOtouch™ RESP system (Fukuda Colin Co., Ltd., Tokyo, Japan). Electrocardiogram electrodes were attached bilaterally below the clavicles. The system enables continuous cuffless blood pressure measurement based on pulse transit time. This pulse transit time-based blood pressure measurement method has previously been clinically validated using the SOMNOtouch™ NIBP system [26]. Before recording, the system was individually calibrated for each participant using an initial cuff-based blood pressure measurement. A Biosignalsplux system (PLUX Wireless Biosignals, Lisbon, Portugal) was used to acquire TMP and EDA. EDA electrodes were attached to the palmar surfaces of the left index and middle fingers, and the TMP sensor was fixed to the fingertip of the left middle finger. The sensors were attached to the skin and secured with medical adhesive tape covering them. A sound sensor was positioned near the participant’s ear to record environmental noise. The SOMNOtouch™ RESP system and Biosignalsplux system were synchronized before data acquisition.

### 2.4. Subjective Assessments

Before the experiment, participants practiced using the real-time subjective rating device. During the experiment, participants continuously recorded their perceived discomfort by moving the lever of the device, which they held in their right hand, between the minimum position (no discomfort) and the maximum position (maximum discomfort). The lever position was recorded as a voltage signal and converted to a percentage of the full movement range, ranging from 0% (minimum position; no discomfort) to 100% (maximum position; maximum discomfort). This continuous subjective rating is referred to as real-time discomfort (RTD). The device was connected to the Biosignalsplux system, allowing the RTD signal to be recorded synchronously with the physiological signals.

Subjective assessments also included the State–Trait Anxiety Inventory (STAI) and a Visual Analog Scale (VAS). The STAI is a questionnaire designed to assess both state and trait anxiety using two 20-item scales [27]. State anxiety reflects a transient psychological condition that includes not only anxiety but also feelings of tension, calmness, and reassurance, whereas trait anxiety reflects an individual’s relatively stable tendency to experience anxiety. State anxiety was assessed before and after the experiment, while trait anxiety was assessed once before the experiment. In addition, a 100-mm VAS was used to assess pain, discomfort, fear, anxiety, and overall distress before and after treatment. The post-experiment STAI state anxiety assessment and VAS ratings were based on participants’ retrospective evaluations of their experiences during the experiment. Unlike the 20-item STAI state anxiety scale, which uses a 4-point rating scale (1–4) for each item and yields a total score ranging from 20 to 80, anxiety on the VAS was assessed using a single-item scale ranging from 0 to 100, reflecting the participant’s self-perceived anxiety. 

### 2.5. Data Processing and Feature Extraction

All data were exported in Excel format and processed using Python (version 3.13.9; Anaconda distribution, Jupyter Notebook version 7.4.5). Signals were initially acquired at 1000 Hz and subsequently downsampled to 20 Hz for analysis by retaining every 50th sample. No additional filtering or smoothing was applied to the signals. Before analysis, all continuous waveforms were visually inspected to confirm adequate signal acquisition. Recordings in which the signal remained essentially flat without observable physiological fluctuations were regarded as inadequately recorded and were excluded from the analyses involving EDA or TMP. Short-term fluctuations observed during treatment were retained rather than uniformly classified as artifacts because they could reflect physiological fluctuations associated with the actual dental procedure. For each physiological parameter, the mean value during the final 10 s of Phase A was used as the baseline, and baseline-corrected changes (Δ values) were calculated. The final 10 s was selected to minimize the influence of initial physiological fluctuations after sensor attachment and to obtain a stable baseline at the end of Phase A, immediately before exposure to the experimental stimuli.

For TMP, the mean change during each phase, minimum temperature change, time to minimum temperature, and post-treatment maximum ΔTMP and its occurrence time were calculated. For EDA, the mean change (Mean ΔEDA), maximum change (Maximum ΔEDA), and time to the maximum response were calculated for each phase. For the correlation analysis with trait anxiety, the time to maximum ΔEDA was defined separately for the Treatment and Post-treatment phases as the elapsed time from the beginning of each phase to the occurrence of its maximum ΔEDA. For RTD, the maximum value (RTDmax) and area under the curve (RTD AUC) were calculated separately for the Noise-only and Treatment phases. RTD AUC was calculated by integrating the RTD signal over the entire duration of each phase, without baseline subtraction. RTD values during the baseline rest phase were regarded as reflecting the discomfort actually experienced at that time, rather than as an offset requiring correction.

### 2.6. Statistical Analysis

For phase-averaged physiological changes, differences from baseline (Δ = 0) were assessed using the Wilcoxon signed-rank test with Holm correction for multiple comparisons. For temporal TMP and EDA parameters, differences from baseline (Δ = 0) were assessed using Wilcoxon signed-rank tests with Holm correction for multiple comparisons. Inter-phase differences in TMP and EDA parameters across the three phases were evaluated using the Friedman test, followed by pairwise Wilcoxon signed-rank tests with Holm correction for multiple comparisons. Changes in STAI-state and VAS subjective scores before and after treatment were evaluated using the Wilcoxon signed-rank test. Associations among subjective ratings, RTD, and physiological parameters were assessed using Spearman’s rank correlation coefficient (Spearman’s ρ). Spearman’s ρ was reported as an effect-size measure, together with 95% confidence intervals (CIs), which were estimated using 10,000 bootstrap resamples with the percentile method. *p* values for correlation analyses were adjusted for multiple comparisons using the Holm method within each set of comparisons. These correlation analyses were exploratory. Statistical significance was set at *p* < 0.05.

## 3. Results

### 3.1. Temporal Changes in Subjective Discomfort and Physiological Responses Across Experimental Phases

**Figure 2:**
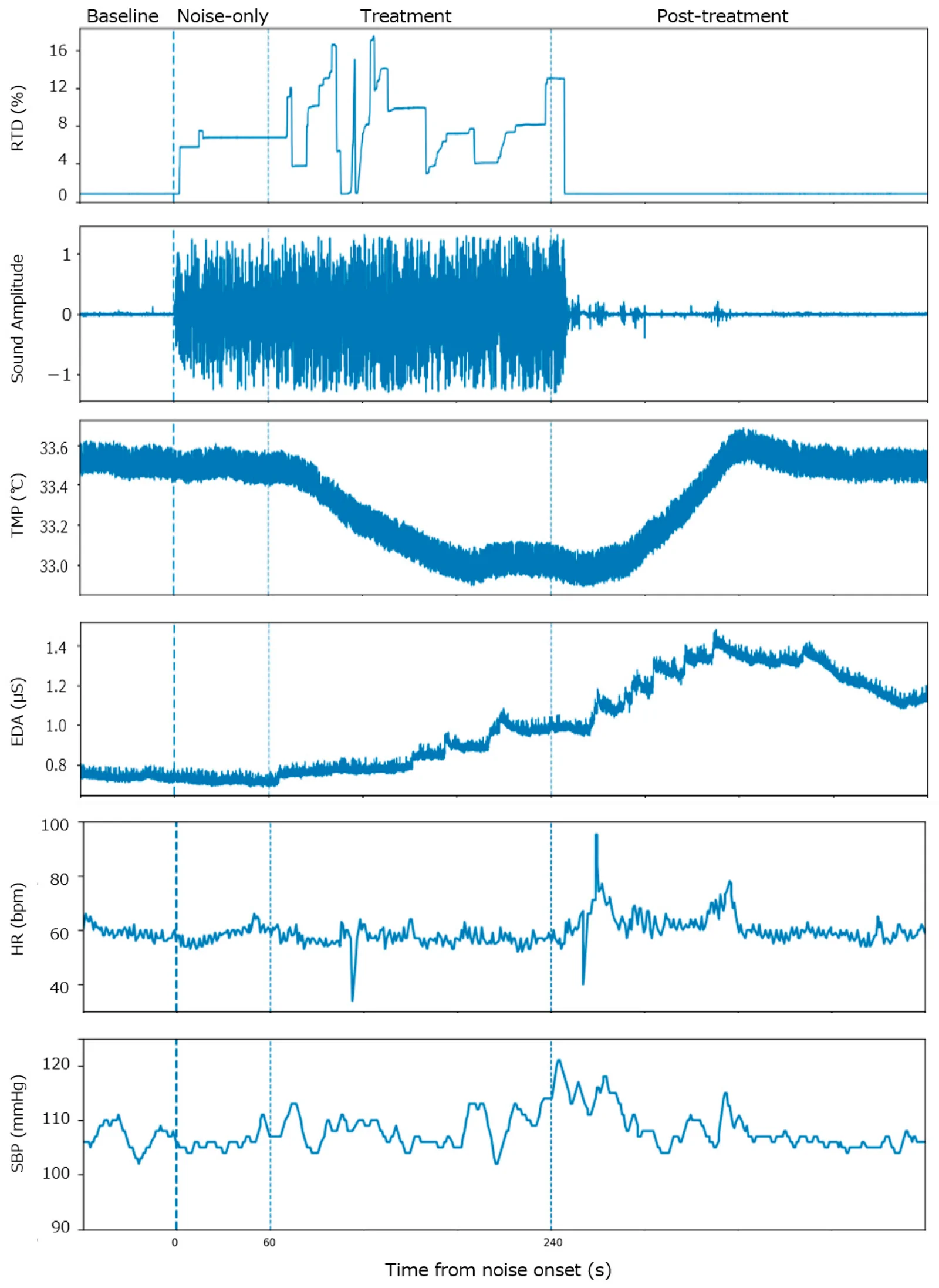
Example of temporal changes in subjective discomfort and physiological responses in one participant. The panels show subjective discomfort (RTD), sound waveform (sound amplitude), fingertip skin temperature (TMP), electrodermal activity (EDA), heart rate (HR), and systolic blood pressure (SBP). Vertical dashed lines indicate the onset of the Noise-only phase (0 s), Treatment phase (60 s), and Post-treatment phase (240 s). Only the final 60 s of Phase A is shown. RTD is expressed as a percentage of the full movement range of the lever (0%, no discomfort; 100%, maximum discomfort).

Figure 2 shows an example of the temporal changes in RTD, TMP, EDA, HR, and SBP, together with the sound waveform, in one participant. In this participant, RTD increased during the Noise-only and Treatment phases but rapidly decreased after treatment completion, with no reported discomfort after the first few seconds of the Post-treatment phase. TMP gradually decreased during treatment, reached its minimum after treatment completion, and subsequently recovered. EDA gradually increased during treatment, continued to increase during the Post-treatment phase, and subsequently decreased. HR showed transient fluctuations during the Noise-only and Treatment phases but remained relatively stable overall. However, relatively large transient fluctuations in HR and SBP were observed shortly after treatment completion, around the time when RTD decreased. Figure S1 shows the TMP time series of the same participant over an extended time window before, during, and after the experimental protocol. The trace illustrates TMP fluctuations outside the experimental period, as well as the decrease during treatment and the subsequent recovery after treatment completion.

Figure S2 shows the temporal changes in RTD and physiological parameters for individual participants (RTD, HR, and SBP: n = 14; TMP and EDA: n = 12). RTD responses varied considerably among participants. Some participants showed clear increases in RTD during the Noise-only and Treatment phases, whereas others reported little discomfort. RTD responses were also observed in some participants during the Baseline phase. In contrast, during the Post-treatment phase, RTD indicated no discomfort in any participant, except during the first few seconds immediately after treatment completion.

Although the magnitude of the decrease and the timing of the minimum value varied among participants, TMP decreased during the Treatment phase in all participants. In most participants, the decrease continued after treatment completion, with the minimum TMP occurring during the Post-treatment phase. In addition, all but one participant showed a recovery trend after reaching the minimum TMP during the Post-treatment phase. EDA also showed considerable inter-individual variability in both response magnitude and temporal course. In some participants, temporal changes were not readily apparent at the scale used in Figure S2; however, closer inspection of the individual traces revealed gradual changes over time, as illustrated in Figure 2.

For HR, relatively large transient fluctuations were observed in several participants during the Post-treatment phase; however, no clear temporal pattern common to all participants was observed during the Treatment phase. For SBP, fluctuations around the onset of the Noise-only or Treatment phase were observed in some participants; however, no clear temporal pattern with a consistent direction was evident across participants. Temporal changes across the experimental phases were more clearly observed in TMP and EDA than in HR and SBP.

Overall, TMP and EDA showed temporal changes during and after treatment, although the magnitude, timing, and recovery patterns varied among participants. To quantitatively characterize these temporal changes and inter-individual differences, multiple temporal features of TMP and EDA were evaluated, as described in the following sections.

### 3.2. Temporal Characteristics of TMP Responses

To quantitatively evaluate the temporal changes in TMP, the minimum change in TMP (minimum ΔTMP) and its timing were analyzed in addition to the mean change (Mean ΔTMP) during each phase (Table 1).

Mean ΔTMP was −0.08 ± 0.05 °C during the Noise-only phase, −0.47 ± 0.20 °C during the Treatment phase, and −0.45 ± 0.42 °C during the Post-treatment phase, all of which were significantly lower than baseline (*p* < 0.01). During the Post-treatment phase, Mean ΔTMP was similar to that during the Treatment phase. In contrast, minimum ΔTMP was −0.30 ± 0.17 °C during the Noise-only phase, −0.86 ± 0.36 °C during the Treatment phase, and −0.99 ± 0.38 °C during the Post-treatment phase. Minimum ΔTMP during the Noise-only phase was significantly lower than baseline (*p* < 0.05). Minimum ΔTMP during the Treatment phase was significantly lower than that during the Noise-only phase (*p* < 0.05). Minimum ΔTMP during the Post-treatment phase was significantly lower than that during the Treatment phase (*p* < 0.05). After reaching its minimum, TMP gradually recovered. To further characterize the recovery after the minimum TMP was reached, the maximum ΔTMP during the Post-treatment phase and its timing were examined. The maximum ΔTMP from baseline observed during the Post-treatment phase was −0.03 ± 0.56 °C and occurred at a mean of 394.2 s (range, 346–475 s) after noise onset.

Thus, the phase-averaged Mean ΔTMP did not capture the within-phase temporal pattern of continued decrease to the minimum followed by recovery during the Post-treatment phase.

### 3.3. Temporal Characteristics of EDA Responses

To quantitatively evaluate the temporal changes in EDA, the maximum change in EDA (Maximum ΔEDA) and its timing were analyzed in addition to the mean change (Mean ΔEDA) during each phase (Table 2).

Mean ΔEDA was significantly higher during the Post-treatment phase (2.38 ± 2.10 μS) than at baseline and during the Noise-only phase (*p* < 0.05), whereas no significant differences from baseline were observed during the Noise-only or Treatment phases. The median Maximum ΔEDA increased from 0.36 μS during the Noise-only phase to 2.13 μS during the Treatment phase and 3.56 μS during the Post-treatment phase. Maximum ΔEDA was significantly greater than baseline in all phases (*p* < 0.05). Maximum ΔEDA was also significantly greater during the Treatment and Post-treatment phases than during the Noise-only phase and during the Post-treatment phase than during the Treatment phase (all *p* < 0.05). The mean occurrence times of Maximum ΔEDA were 27.6 s, 125.9 s, and 319.1 s from noise onset during the Noise-only, Treatment, and Post-treatment phases, respectively.

Overall, Maximum ΔEDA captured EDA responses even in phases in which Mean ΔEDA did not show a significant change. Individual EDA waveforms showed inter-individual differences in response timing, magnitude, and persistence (Figure S2).

### 3.4. Psychological Correlates of EDA Responses

Given the inter-individual variability in EDA responses, the relationships between trait anxiety and EDA response characteristics were examined. Trait anxiety was significantly positively correlated with the time to Maximum ΔEDA during the Treatment phase (ρ = 0.69, 95% CI: 0.27 to 0.92, Holm-adjusted *p* = 0.025), whereas no significant correlation was observed during the Post-treatment phase (ρ = −0.06, 95% CI: −0.67 to 0.63, Holm-adjusted *p* = 0.846) (Table 3).

### 3.5. Relationships Between RTD and Physiological Responses

As RTD responses were observed predominantly during the Treatment phase, the relationships between Treatment-phase RTD measures and physiological responses were examined (Table 4). No significant correlations were observed between Treatment-phase RTDmax or RTD AUC and TMP or EDA responses during the Treatment phase. As physiological responses persisted into the Post-treatment phase, their relationships with Treatment-phase RTD measures were also examined. Treatment-phase RTD AUC showed a negative correlation with maximum ΔTMP during the Post-treatment phase (ρ = −0.66, 95% CI: −0.89 to −0.19), but this association was not statistically significant after Holm correction (adjusted *p* = 0.148). Overall, no correlations remained statistically significant after Holm correction.

### 3.6. Relationships Between Questionnaire Scores and RTD

Correlations between post-treatment questionnaire scores and RTD measures (RTDmax and RTD AUC) are summarized in Table S1. Several correlation coefficients indicated moderate to strong positive associations between questionnaire scores and RTD measures, particularly during the Treatment phase; however, the confidence intervals were generally wide, and none of the correlations remained statistically significant after Holm correction across the 24 correlations examined (six questionnaire measures × four RTD measures). Thus, no specific post-treatment questionnaire measure showed a consistent association with RTD.

The results of the VAS and STAI assessments conducted before and after the experiment are shown in Figure S3. Overall distress, pain, discomfort, and state anxiety significantly increased after treatment (*p* < 0.01), whereas no significant pre–post changes were observed in fear or VAS anxiety. The relationships between retrospective ratings of overall distress and the other subjective measures were also examined. Positive correlations were observed between overall distress and pain (ρ = 0.92, 95% CI: 0.72 to 0.99, Holm-adjusted *p* < 0.001), discomfort (ρ = 0.70, 95% CI: 0.14 to 0.98, Holm-adjusted *p* = 0.011), and VAS anxiety (ρ = 0.80, 95% CI: 0.46 to 0.95, Holm-adjusted *p* = 0.002), whereas the correlation with fear was relatively weak (ρ = 0.36, 95% CI: −0.30 to 0.83, Holm-adjusted *p* = 0.200).

## 4. Discussion

### 4.1. TMP Responses During Dental Treatment

Peripheral skin temperature is influenced by sympathetic vasoconstriction and has been investigated in relation to psychological stress and mental workload [17,20,28]. In the present study, fingertip skin temperature was continuously recorded together with subjective and other physiological measures to characterize responses during dental treatment.

A significant decrease in TMP was observed during the Treatment phase. Importantly, the continuous TMP trajectories were not necessarily monotonic over the course of the experiment. Consequently, phase-averaged values or a single linear temporal trend may not fully capture the temporal characteristics of the response and may obscure information regarding its magnitude, timing, and recovery. To characterize changes in the continuously recorded temperature data that were not represented by phase-averaged values alone, we therefore evaluated the minimum change in TMP and time to minimum. These temporally interpretable features allowed the magnitude and timing of the temperature decrease to be quantified over the course of the experiment.

Notably, responses were also observed during the Noise-only phase, when no intraoral treatment was performed. Some participants showed increases in RTD during this phase, and decreases in TMP were also observed in some participants. Previous studies have reported associations between sound exposure and peripheral physiological responses. Vasoconstrictive responses accompanied by decreased fingertip blood flow have been observed even in response to brief auditory stimuli (500 ms) with a maximum A-weighted sound pressure level of 70 dB [29]. Experimental research using simulated construction noise at levels of up to 85 dB has also shown decreases in fingertip skin temperature and fingertip blood flow following the onset of noise exposure, supporting an association between noise exposure and peripheral vasoconstrictive responses [30]. In addition, dental drill noise and suction noise are perceived as unpleasant and may contribute to anxiety and discomfort in dental settings [12,13,14]. These findings are consistent with the possibility that treatment-related noise may contribute to subjective and peripheral physiological responses even before intraoral treatment begins. During actual dental treatment, however, patients are exposed not only to sound but also to multiple other stimuli, including vibration and intraoral manipulation.

Taken together, continuous fingertip temperature measurement may provide one component of multimodal monitoring of physiological responses during dental treatment. By quantifying the magnitude and timing of temperature changes from continuous recordings, this approach may provide information on peripheral physiological responses that is not fully represented by phase-averaged values alone.

### 4.2. EDA Responses During Dental Treatment

EDA is a well-established physiological indicator of sympathetic nervous system activity and is known to be sensitive to changes in emotional responses and arousal levels [18,19]. In the present study, EDA was continuously recorded together with subjective and other physiological measures to characterize responses during dental treatment. Mean ΔEDA and Maximum ΔEDA showed different patterns across the experimental phases. Although Mean ΔEDA did not differ significantly from baseline during the Treatment phase, Maximum ΔEDA was already significantly greater than baseline during the Noise-only phase and increased further during the Treatment and Post-treatment phases (Table 2). These findings suggest that evaluating EDA responses during dental treatment requires consideration not only of average response levels but also of temporal characteristics, including response magnitude, timing, and persistence.

EDA responses are known to exhibit inter-individual variability, and response latency and recovery may vary across individuals [19]. Consistent with this, inter-individual variability in the temporal course of EDA responses was observed in this study. The individual EDA trajectories shown in Figure S2 illustrate differences in response timing, magnitude, persistence, and recovery among participants. Thus, despite undergoing the same experimental protocol, participants did not exhibit a uniform temporal course of EDA responses. Group averaging may therefore obscure important temporal characteristics of individual EDA responses.

In conventional EDA research, single stimuli or multiple stimuli presented at controlled intervals are commonly used to evaluate skin conductance response (SCR) measures such as amplitude, latency, response frequency, and peak counts [15,19]. In contrast, during dental treatment, different types of stimuli, including noise, vibration, intraoral stimulation, and pain, may occur continuously or repeatedly without controlled intervals. Under such conditions, individual SCRs may overlap over time, making it difficult to clearly distinguish responses to individual stimuli. Therefore, the sustained EDA elevations observed in this study may reflect not only prolonged individual responses but also the temporal summation of repeated SCRs. In dental treatment settings, where individual SCRs may be difficult to separate, analyses that capture the overall temporal course of EDA responses, including response timing, may provide useful information beyond that obtained from analyses of individual SCRs alone.

We next examined whether trait anxiety was associated with the temporal characteristics of EDA responses. Trait anxiety represents a general tendency to experience anxiety across situations rather than anxiety related to a specific situation [27]. In this study, trait anxiety was positively correlated with the time to maximum ΔEDA during the Treatment phase, whereas no significant correlation was observed during the Post-treatment phase (Table 3). This finding suggests a possible association between trait anxiety and the timing of EDA peak responses during treatment. However, given the small sample size and exploratory nature of the analysis, this association should be interpreted cautiously.

Taken together, these findings suggest that continuous EDA measurement may provide one component of multimodal monitoring of physiological responses during dental treatment. EDA responses showed inter-individual variability in their temporal course. Incorporating response magnitude, peak timing, and the overall temporal course may provide a more detailed characterization of EDA responses during dental treatment.

### 4.3. Relationships Between Subjective Evaluations and Physiological Responses

Psychophysiological studies in other fields have used analyses that account for temporal lags when examining relationships between continuous subjective emotional ratings and autonomic responses [31,32,33]. In conventional EDA research, stimulus-related SCRs are typically identified within a latency window of several seconds following clearly defined stimuli, and closely spaced stimuli may result in overlapping responses [34]. Dental treatment, however, represents a more complex setting in which patients may experience anxiety even before treatment begins [35,36,37] and multiple stimuli, including noise, vibration, intraoral stimulation, and pain, may subsequently occur continuously or repeatedly during treatment. As discussed in Section 4.2, the resulting physiological responses may overlap over time. Therefore, the relationship between subjective and physiological responses may not be fully captured by simple comparisons at the same time point or within the same phase, or by assuming a fixed temporal shift between them.

In the present study, subjective discomfort and physiological responses showed different temporal courses. Subjective discomfort recorded by RTD was evident during dental treatment but rapidly decreased after treatment completion, with little response observed during the Post-treatment phase, whereas TMP and EDA responses persisted into the Post-treatment phase. In this context, a negative correlation was observed between Treatment-phase RTD AUC and Post-treatment maximum ΔTMP (ρ = −0.66), although this association did not remain statistically significant after correction for multiple comparisons (Holm-adjusted *p* = 0.148; Table 4). Although this exploratory finding does not establish an association between subjective discomfort and subsequent TMP responses, it provides a basis for further consideration of their temporal relationship.

Overall distress assessed retrospectively after treatment was positively associated with pain, discomfort, and VAS anxiety, suggesting that this global rating may reflect multiple aspects of the subjective experience rather than a single specific sensation. Post-treatment subjective ratings provide retrospective evaluations of the treatment experience but cannot directly capture how discomfort changes over the course of treatment. In contrast, continuous RTD recording provides information on changes in discomfort over time, with RTDmax representing the maximum intensity of discomfort and RTD AUC reflecting both the intensity and duration of discomfort. Although moderate to strong correlations were observed between some subjective ratings retrospectively assessed after treatment and RTD measures, the confidence intervals were generally wide, and none remained statistically significant after correction for multiple comparisons (Table S1). These findings do not indicate that either retrospective or real-time assessment is superior; rather, the two approaches may capture different aspects of the subjective treatment experience, particularly with respect to its temporal course and retrospective evaluation.

Taken together, these findings highlight the importance of considering not only response magnitude but also the temporal course of responses, including their timing, persistence, and recovery, when examining relationships between subjective and physiological responses during dental treatment. Both the timing of subjective assessment and the temporal characteristics of physiological responses should therefore be considered when interpreting their relationships.

### 4.4. Limitations and Future Directions

This study has some limitations. First, this pilot study had a limited sample size, with imbalances in the sex and age distributions. Accordingly, the observed temporal characteristics and relationships should be regarded as exploratory findings. Future studies with larger and more diverse clinical populations are needed to examine the potential influence of participant characteristics, such as age and sex, on physiological responses. Second, the experimental phases were conducted in a fixed sequence to reflect the temporal progression of a clinical dental procedure, and no separate rest-only control condition was included. While this design allowed physiological responses to be continuously examined from pre-treatment rest through treatment to the post-treatment period, the contribution of time-dependent physiological changes could not be completely separated from stimulus-related responses. Room temperature was not recorded during the experiment, preventing a quantitative assessment of its potential contribution to the observed TMP changes. Consequently, thermoneutral conditions throughout the experimental protocol could not be objectively confirmed, and a possible contribution of time-dependent thermal drift cannot be completely excluded. Accordingly, the effects of individual phases or stimuli should not be interpreted as independent causal effects. Third, this study focused on ultrasonic scaling, a minimally invasive dental procedure, and the magnitude of subjective discomfort measured by RTD varied among participants. Whether subjective and physiological responses would differ during more invasive procedures, such as treatments involving local anesthesia, tooth preparation, or oral surgical procedures, remains unclear.

HR and SBP are also important cardiovascular measures for assessing physiological responses during dental treatment and were continuously recorded in the present study. Overall, changes in HR and SBP did not show consistent temporal characteristics in relation to the experimental phases or treatment-related stimuli across participants, in contrast to those observed for TMP and EDA. Detailed temporal characterization of HR and SBP was therefore not performed in the present study. The characteristics of the pulse transit time-based blood pressure measurement method should also be considered when interpreting short-term changes in SBP [26].

Cardiovascular fluctuations may also be influenced by factors specific to dental treatment, including changes in swallowing and breathing and restriction of body movement associated with maintaining an open-mouth position while temporarily retaining water in the oral cavity during procedures involving water irrigation. Further investigation is needed to characterize the influence of these factors and the temporal characteristics of cardiovascular responses during dental treatment.

Recent studies have increasingly applied machine-learning approaches to physiological signals obtained from wearable sensors to estimate states such as stress, pain, and anxiety [38,39]. However, predictive importance does not by itself establish the physiological or clinical meaning of an input feature. Before features derived from physiological signals are used in statistical or predictive models, it is important to examine whether their quantitative representation adequately reflects the temporal characteristics of the underlying physiological response. The present findings illustrate this point: phase-averaged values provide one quantitative representation of physiological changes, whereas continuous recordings additionally allow response magnitude, peak timing, persistence, and recovery to be characterized. Thus, how continuous physiological signals are quantified may influence which aspects of the physiological response are represented in subsequent analyses.

Future studies with larger datasets should therefore first examine the physiological interpretation of quantitatively derived temporal features and then consider their incorporation into statistical or predictive models. Longitudinal analyses using nonlinear or piecewise mixed-effects models that account for serial autocorrelation may also be useful for further characterizing these temporal responses. To extend such approaches to real-world dental settings, where multiple stimuli change over time, it will be important to consider the persistence and recovery of physiological responses and their temporal relationships with subjective responses. Incorporating these temporal characteristics together with continuous subjective ratings may contribute to the development of physiologically interpretable real-time monitoring and predictive models using wearable physiological sensors.

As a longer-term goal, such physiologically interpretable monitoring approaches may be applicable to patients who have difficulty expressing their subjective states during treatment, including children, older adults, patients with dental anxiety, and individuals with disabilities.

## 5. Conclusions

In this study, physiological responses, including TMP and EDA, were measured simultaneously with RTD to characterize physiological and subjective responses during dental treatment. Their relationships with subjective evaluations and trait anxiety were also examined. Analyses of the magnitude, timing, persistence, and recovery of physiological responses revealed temporal response characteristics and inter-individual differences not fully captured by mean-value analyses alone, including differences in response timing and sustained physiological responses. Continuous RTD recording also captured changes in subjective discomfort over the course of treatment.

Overall, the findings suggest that physiological and subjective responses during dental treatment may not be fully characterized by mean physiological values or post-treatment subjective evaluations alone. Incorporating the temporal characteristics of physiological responses together with continuous subjective ratings may provide a more detailed characterization of patient responses during dental treatment. Importantly, the findings highlight the need to consider whether quantitative features derived from continuous physiological signals adequately represent the temporal characteristics of the underlying responses before these features are used in subsequent statistical or predictive analyses.

## Figures and Tables

**Table 1:** Phase-averaged and minimum TMP changes during the experimental phases.

Parameter	Noise-Only	Treatment	Post-Treatment
Mean ΔTMP from baseline, mean ± SD (°C)	−0.08 ± 0.05 **	−0.47 ± 0.20 **	−0.45 ± 0.42 **
Minimum TMP change from baseline, mean ± SD (°C)	−0.30 ± 0.17 *	−0.86 ± 0.36 *^†^	−0.99 ± 0.38 *^†‡^
Time to minimum TMP from noise onset, mean (range), s	56.1 (42–60)	223.9 (154–240)	293.1 (270–411)

* *p* < 0.05 vs. baseline; ** *p* < 0.01 vs. baseline; ^†^ *p* < 0.05 vs. Noise-only minimum TMP; ^‡^ *p* < 0.05 vs. Treatment minimum TMP. Comparisons against baseline (Δ = 0) were performed using Wilcoxon signed-rank tests with Holm correction for multiple comparisons. Differences in minimum TMP among the Noise-only, Treatment, and Post-treatment phases were assessed using the Friedman test, followed by pairwise Wilcoxon signed-rank tests with Holm correction for multiple comparisons when significant. TMP: fingertip skin temperature; SD: standard deviation.

**Table 2:** Temporal characteristics of EDA responses during the Noise-only, Treatment, and Post-treatment phases.

Parameter	Noise-Only	Treatment	Post-Treatment
Mean ΔEDA from baseline, mean ± SD (μS)	0.16 ± 0.30	1.25 ± 1.99	2.38 ± 2.10 *^†^
Maximum ΔEDA, median (range) (μS)	0.36 * (0.05–1.83)	2.13 *^†^ (−0.04 to 12.02)	3.56 *^†‡^ (0.74–15.50)
Time to Maximum ΔEDA, mean (range) (s)	27.6 (1–57)	125.9 (62–232)	319.1 (265–421)

* *p* < 0.05 vs. baseline; ^†^ *p* < 0.05 vs. Noise-only phase; ^‡^ *p* < 0.05 vs. Treatment phase. Time values indicate the occurrence time of the phase-specific Maximum ΔEDA from noise onset. Comparisons against baseline (Δ = 0) were performed using Wilcoxon signed-rank tests with Holm correction for multiple comparisons. Differences among the Noise-only, Treatment, and Post-treatment phases were assessed using the Friedman test, followed by pairwise Wilcoxon signed-rank tests with Holm correction for multiple comparisons when significant. EDA: electrodermal activity; SD: standard deviation.

**Table 3:** Correlations between trait anxiety and temporal characteristics of EDA responses.

Questionnaire Measure	EDA Temporal Characteristic	ρ (95% CI)	Holm-Adjusted *p*
Trait anxiety	Time to Maximum ΔEDA (Treatment)	0.69 (0.27, 0.92)	0.025 *
Trait anxiety	Time to Maximum ΔEDA (Post-treatment)	−0.06 (−0.67, 0.63)	0.846

Spearman’s rank correlation coefficients (ρ) with 95% confidence intervals (CIs) are shown. CIs were estimated using 10,000 bootstrap resamples with the percentile method. *p* values were adjusted for the two phase-specific comparisons using the Holm method. All analyses were based on n = 12. * *p* < 0.05 after Holm correction. EDA: electrodermal activity.

**Table 4:** Correlations between RTD ratings and physiological responses.

RTD Measure	Physiological Measure	ρ (95% CI)	Holm-Adjusted *p* Value
RTDmax (Treatment)	Treatment minimum ΔTMP	0.31 (−0.43, 0.83)	1.000
	Treatment maximum ΔEDA	0.27 (−0.45, 0.87)	1.000
	Post-treatment maximum ΔTMP	−0.34 (−0.75, 0.26)	1.000
	Post-treatment maximum ΔEDA	0.14 (−0.44, 0.68)	1.000
RTD AUC (Treatment)	Treatment minimum ΔTMP	0.14 (−0.60, 0.75)	1.000
	Treatment maximum ΔEDA	0.40 (−0.26, 0.85)	1.000
	Post-treatment maximum ΔTMP	−0.66 (−0.89, −0.19)	0.148
	Post-treatment maximum ΔEDA	0.13 (−0.49, 0.68)	1.000

Values indicate Spearman’s rank correlation coefficients (ρ) with 95% confidence intervals (CIs) in parentheses. CIs were estimated using 10,000 bootstrap resamples with the percentile method. *p* values were adjusted for eight comparisons using the Holm method. All analyses were based on n = 12. RTD: real-time discomfort; AUC: area under the curve; TMP: fingertip skin temperature; EDA: electrodermal activity.

## Data Availability

The data are not publicly available due to privacy and ethical restrictions.

## Supplementary Materials

The following supporting information can be downloaded at https://www.mdpi.com/article/10.3390/s26216695/s1. Figure S1: TMP time series over an extended time window before, during, and after the experimental protocol; Figure S2: Individual temporal changes in subjective discomfort and physiological responses during the experimental protocol; Figure S3: Changes in subjective ratings before and after the experiment; Table S1: Correlations between questionnaire scores and RTD ratings.

## Abbreviations

The following abbreviations are used in this manuscript:

AUC	Area under the curve
CI	Confidence interval
EDA	Electrodermal activity
HR	Heart rate
*L*_Aeq_	A-weighted equivalent continuous sound pressure level
Maximum ΔEDA	Maximum change in EDA
Mean ΔEDA	Mean change in EDA
RTD	Real-time discomfort
RTD AUC	AUC of the RTD signal
RTDmax	Maximum real-time discomfort
SBP	Systolic blood pressure
SCR	Skin conductance response
SD	Standard deviation
STAI	State–Trait Anxiety Inventory
TMP	Fingertip skin temperature
VAS	Visual Analog Scale

## References

[1] Oosterink F.M.D., de Jongh A., Hoogstraten J. (2009). Prevalence of dental fear and phobia relative to other fear and phobia subtypes. Eur. J. Oral Sci..

[2] Silveira E.R., Cademartori M.G., Schuch H.S., Armfield J.M., Demarco F.F. (2021). Estimated prevalence of dental fear in adults: A systematic review and meta-analysis. J. Dent..

[3] Hutse I., Coppens M., Herbelet S., Seyssens L., Marks L. (2021). Syncope in dental practices: A systematic review on aetiology and management. J. Evid. Based Dent. Pract..

[4] Matsumura K., Miura K., Takata Y., Kurokawa H., Kajiyama M., Abe I., Fujishima M. (1998). Changes in blood pressure and heart rate variability during dental surgery. Am. J. Hypertens..

[5] Speirs R.L., Barsby M.J. (1995). Hyperventilation in the dental chair. Dent. Update.

[6] Ho J.C.Y., Chai H.H., Lo E.C.M., Huang M.Z., Chu C.H. (2024). Strategies for effective dentist–patient communication: A literature review. Patient Prefer. Adherence.

[7] Ho J.C.Y., Chai H.H., Luo B.W., Lo E.C.M., Huang M.Z., Chu C.H. (2025). An overview of dentist–patient communication in quality dental care. Dent. J..

[8] Loo C.Y., Graham R.M., Hughes C.V. (2009). Behaviour guidance in dental treatment of patients with autism spectrum disorder. Int. J. Paediatr. Dent..

[9] Aguirre-González G., de Ávila-Rojas P., García-Flores R., Ruiz-Rodríguez R., Pozos-Guillén A., Garrocho-Rangel A. (2020). Inclusive dentistry: Integral management of pediatric patients with intellectual disability and/or communication impairments. Case-series reports. J. Clin. Pediatr. Dent..

[10] Tang S.-J., Wei H.-L., Li C.-Y., Huang M.-N. (2023). Management strategies of dental anxiety and uncooperative behaviors in children with autism spectrum disorder. BMC Pediatr..

[11] Kildahl A.N., Berggren T., Rønneberg A., Molnes S.C.Y., Nordgarden H. (2024). A call for trauma-informed dental care for individuals with intellectual disabilities. Spec. Care Dent..

[12] Yamada T., Kuwano S., Ebisu S., Hayashi M. (2016). Statistical analysis for subjective and objective evaluations of dental drill sounds. PLoS ONE.

[13] Yamada T., Kuwano S., Ebisu S., Hayashi M. (2021). Effect of processing of dental drill noise on subjective impression. Appl. Acoust..

[14] Yamada T., Nozaki K., Hayashi M., Kuwano S. (2023). Sound environment during dental treatment in relation to COVID-19 pandemic. Acoustics.

[15] Bari D.S., Rammoo M.N.S., Youssif A.A., Najman H.M., Aldosky H.Y.Y., Tronstad C., Hou J., Martinsen Ø.G. (2024). Electrodermal activity for quantitative assessment of dental anxiety. J. Sens. Actuator Netw..

[16] Shu L., Xie J., Yang M., Li Z., Li Z., Liao D., Xu X., Yang X. (2018). A review of emotion recognition using physiological signals. Sensors.

[17] Kreibig S.D. (2010). Autonomic nervous system activity in emotion: A review. Biol. Psychol..

[18] Critchley H.D. (2002). Electrodermal responses: What happens in the brain. Neuroscientist.

[19] Boucsein W. (2012). Electrodermal Activity.

[20] Kistler A., Mariauzouls C., von Berlepsch K. (1998). Fingertip temperature as an indicator for sympathetic responses. Int. J. Psychophysiol..

[21] Sharma A., Pant R., Priyadarshi S., Agarwal N., Tripathi S., Chaudhary M. (2019). Cardiovascular changes due to dental anxiety during local anesthesia injection for extraction. J. Maxillofac. Oral Surg..

[22] Huang J., Wu Y., Li F., Zhang D., Tan S. (2026). Assessing pulse rate variability from a wrist-worn PPG device against ECG-derived heart rate variability in ambulatory settings. Smart Wearable Technol..

[23] Gasparro R., Leonetti G., Riccio M., Irace A., Sammartino G., Blasi A., Scandurra C., Maldonato N.M., Sammartino P., Marenzi G. (2021). Thermography as a method to detect dental anxiety in oral surgery. Appl. Sci..

[24] Gomes H.S., Anabuki A.A., Viana K.A., Abreu L.G., Batista A.C., Hosey M.T., Costa L.R. (2022). Assessment of child’s dental anxiety/fear and stress during dental treatment: A systematic review by CEDACORE. Braz. Oral Res..

[25] Ioannou S., Gallese V., Merla A. (2014). Thermal infrared imaging in psychophysiology: Potentialities and limits. Psychophysiology.

[26] Bilo G., Zorzi C., Ochoa Munera J.E., Torlasco C., Giuli V., Parati G. (2015). Validation of the Somnotouch-NIBP noninvasive continuous blood pressure monitor according to the European Society of Hypertension International Protocol revision 2010. Blood Press. Monit..

[27] Spielberger C.D. (1983). Manual for the State-Trait Anxiety Inventory (STAI).

[28] Engert V., Merla A., Grant J.A., Cardone D., Tusche A., Singer T. (2014). Exploring the use of thermal infrared imaging in human stress research. PLoS ONE.

[29] Sato T.G., Ooishi Y. (2012). Sound presentation during different respiration phases alters the sound-induced vasoconstriction mediated by the sympathetic nerve. Neurosci. Lett..

[30] Sugawara K., Takeno E., Mabuchi T., Sukeda A., Ohashi S., Ohnishi N. (2024). Effects of hand-bathing on noise-induced vasoconstriction: A randomized controlled trial. Jpn. J. Nurs. Sci..

[31] Brown C.L., Van Doren N., Ford B.Q., Mauss I.B., Sze J.W., Levenson R.W. (2020). Coherence between subjective experience and physiology in emotion: Individual differences and implications for well-being. Emotion.

[32] Sato W., Kochiyama T., Yoshikawa S. (2020). Physiological correlates of subjective emotional valence and arousal dynamics while viewing films. Biol. Psychol..

[33] Meijer A.L., Arts L.P.A., Gomez R., van den Broek E.L. (2023). Electrodermal activity: A continuous monitor of well-being. J. Smart Cities Soc..

[34] Boucsein W., Fowles D.C., Grimnes S., Ben-Shakhar G., Roth W.T., Dawson M.E., Filion D.L. (2012). Publication recommendations for electrodermal measurements. Psychophysiology.

[35] Lundberg A., Srinivasan M. (2021). Effect of the presence of an aquarium in the waiting area on the stress, anxiety and mood of adult dental patients: A controlled clinical trial. PLoS ONE.

[36] Jackson C., Lindsay S. (1995). Reducing anxiety in new dental patients by means of leaflets. Br. Dent. J..

[37] Lodge J., Tripp G. (1993). Dental students’ perception of patient anxiety. N. Z. Dent. J..

[38] Peitzsch A.G., Geary K., Kong Y., Posada-Quintero H., Havard D., D’Angelo W.R., Chon K.H. (2026). Multimodal detection of pain and anticipation anxiety from ultra-short duration wearable sensors measurements. Sensors.

[39] Moser M.K., Ehrhart M., Resch B. (2024). An explainable deep learning approach for stress detection in wearable sensor measurements. Sensors.

