# Supplementary material for: Real-Time Subjective and Physiological Responses During Dental Treatment: Temporal Characteristics of Skin Temperature and Electrodermal Activity
Source: Sensors (Basel). 2026 Oct 23;26(21):6695. doi: 10.3390/s26216695 (PMC13765573; doi:10.3390/s26216695)
Supplement: Supplementary file 1 [file sensors-26-06695-s001.zip › sensors-4568330-supplementary.pdf]

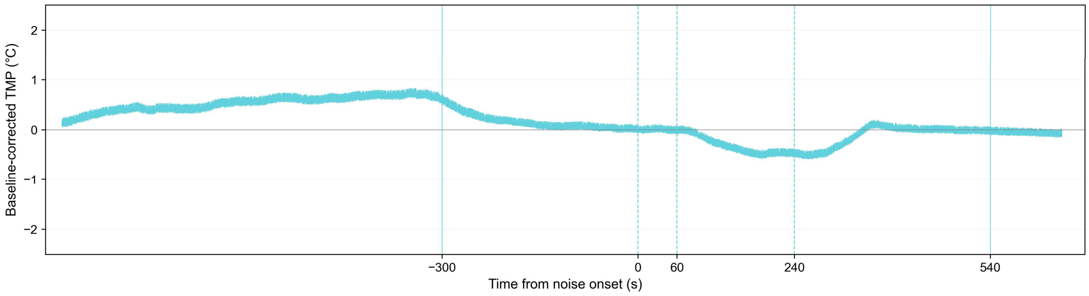

Figure S1. TMP time series of the participant shown in Figure 2 over an extended time window before, during, and after the experimental protocol. TMP values are expressed as changes from the mean temperature during the final 10 s of Phase A. Vertical dashed lines indicate the onset of the Noise-only phase (0 s), Treatment phase (60 s), and Post-treatment phase (240 s), while vertical solid lines indicate the beginning (−300 s) and end (540 s) of the experimental protocol. Before the experimental protocol, the participant was seated in a reclining dental chair. At the beginning of the protocol, the chair was reclined to place the participant in a supine position, which was maintained throughout the protocol. After completion of the protocol, the chair was returned to an upright position. TMP: fingertip skin temperature.

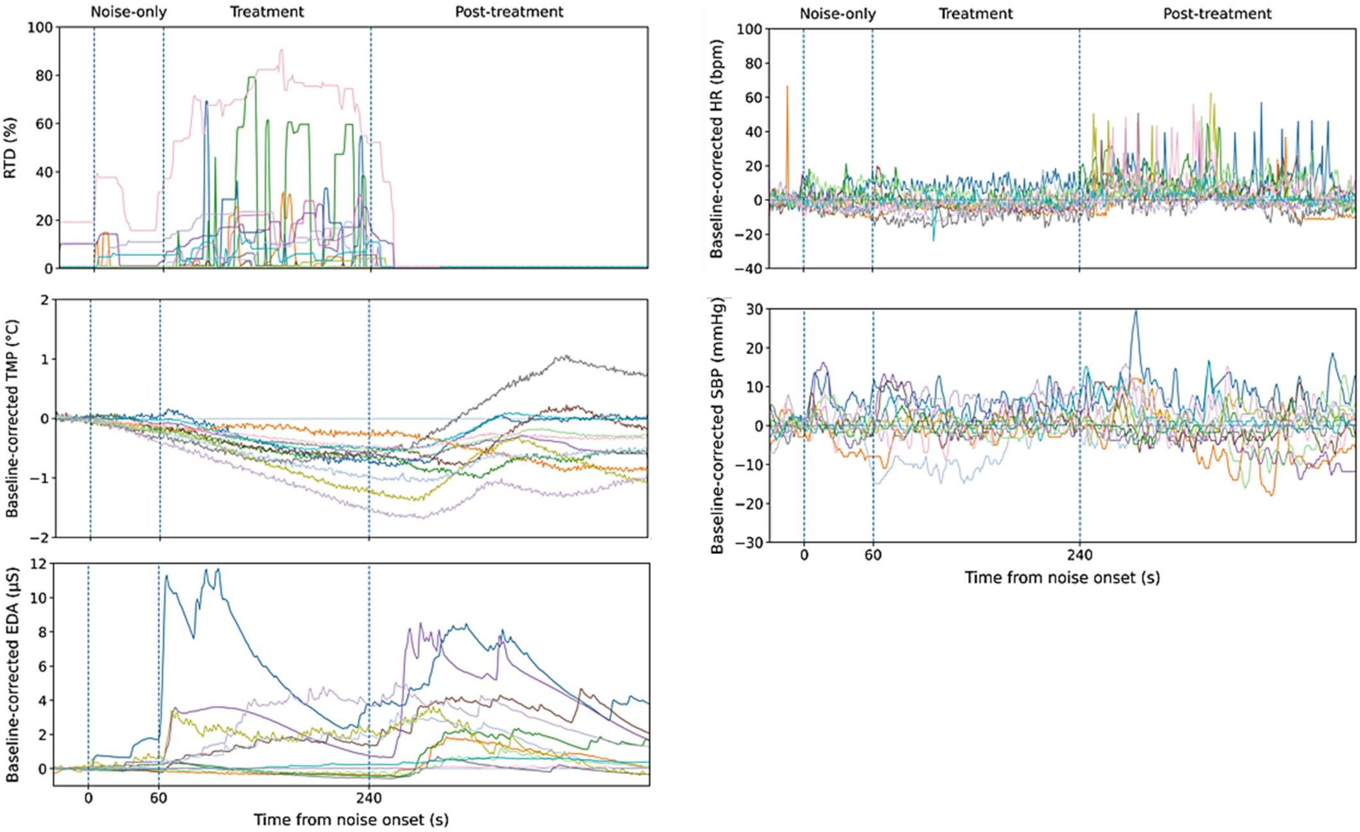

Figure S2. Individual temporal changes in subjective discomfort and physiological responses during the experimental protocol. Individual time-series data are shown for RTD (n = 14), TMP (n = 12), EDA (n = 12), HR (n = 14), and SBP (n = 14). TMP, EDA, HR, and SBP are expressed as changes from each participant's baseline value. The participant shown in Figure 2 and Figure S1 is shown in cyan. Vertical dashed lines indicate the onset of the Noise-only phase (0 s), Treatment phase (60 s), and Post-treatment phase (240 s). RTD: real-time discomfort; TMP: fingertip skin temperature; EDA: electrodermal activity; HR: heart rate; SBP: systolic blood pressure.

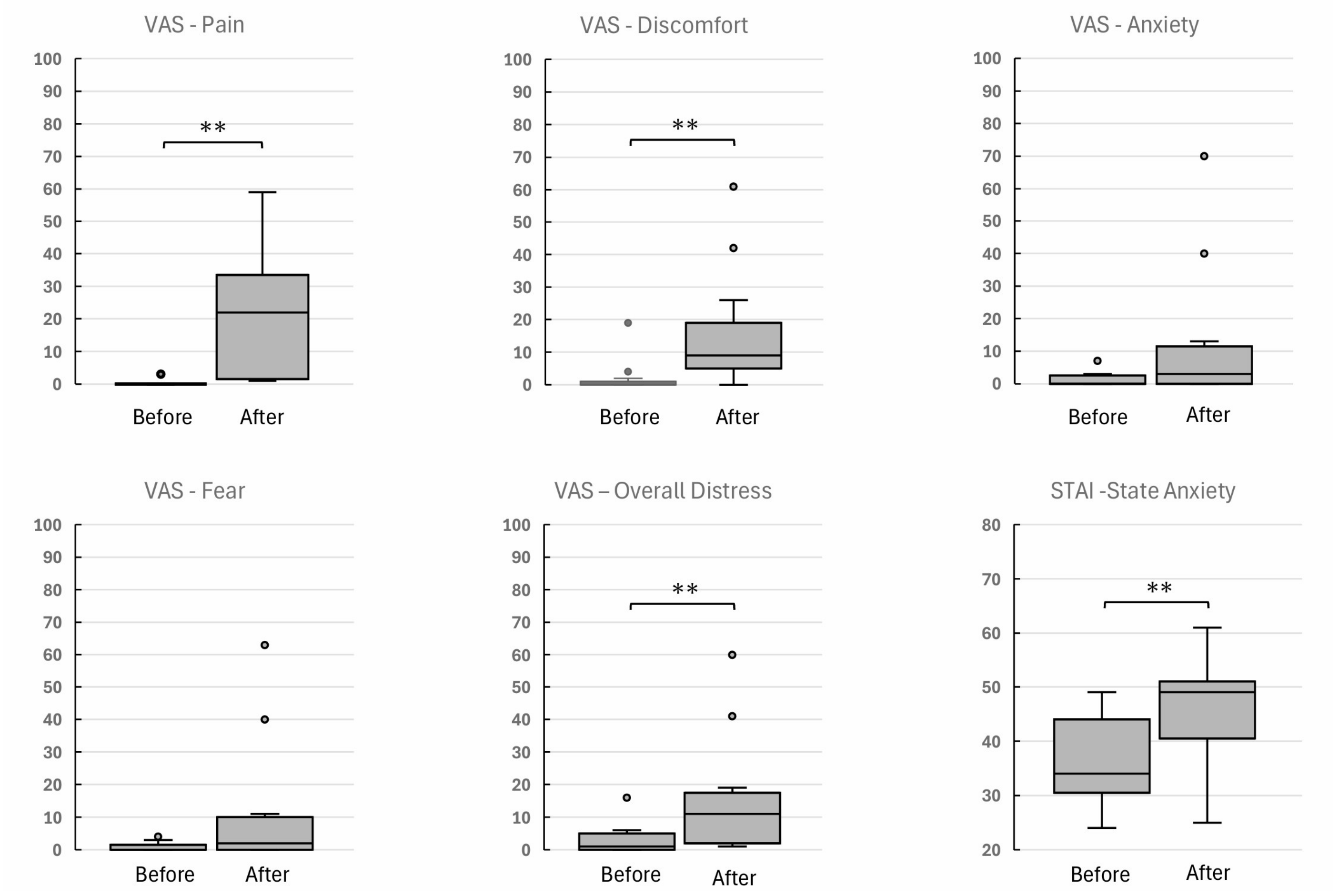

Figure S3. Changes in subjective ratings before and after the experiment. Box plots show VAS scores (0–100) for pain, discomfort, anxiety, fear, and overall distress, and STAI-state anxiety scores (20–80) before and after the experiment. Boxes indicate the interquartile range (25th–75th percentiles), center lines indicate the median, whiskers extend to the most extreme values within 1.5 times the interquartile range, and points beyond the whiskers indicate outliers (n = 14). **$p < 0.01$ vs. before the experiment.
VAS: Visual Analog Scale; STAI: State–Trait Anxiety Inventory.

Table S1. Correlations between questionnaire scores and RTD ratings.

Questionnaire measure (After experiment)	Noise-only		Treatment	
	RTDmax	RTD AUC	RTDmax	RTD AUC
Overall distress	0.62 (0.15, 0.84)	0.65 (0.21, 0.89)	0.48 (−0.18, 0.87)	0.58 (0.00, 0.90)
Pain	0.46 (−0.06, 0.83)	0.50 (−0.04, 0.90)	0.47 (−0.17, 0.75)	0.58 (0.04, 0.85)
Discomfort	0.17 (−0.40, 0.68)	0.21 (−0.42, 0.75)	0.62 (0.12, 0.89)	0.59 (0.05, 0.90)
Fear	−0.05 (−0.68, 0.49)	0.09 (−0.61, 0.62)	0.29 (−0.25, 0.72)	0.45 (−0.11, 0.82)
VAS anxiety	0.38 (−0.16, 0.79)	0.47 (−0.10, 0.87)	0.44 (−0.20, 0.80)	0.58 (0.03, 0.91)
State anxiety	0.19 (−0.43, 0.63)	0.23 (−0.42, 0.71)	0.54 (−0.01, 0.89)	0.57 (0.01, 0.88)

Values indicate Spearman's rank correlation coefficients (ρ) with 95% confidence intervals in parentheses. Confidence intervals were estimated using 10,000 bootstrap resamples (percentile method). P values were adjusted for 24 comparisons using the Holm method; none remained statistically significant after adjustment. All analyses were based on n = 14. RTD: real-time discomfort; AUC: area under the curve; VAS: Visual Analog Scale.
